# Morphology, Behaviour and Evolution of *Gallotia* Lizards from the Canary Islands

**DOI:** 10.3390/ani13142319

**Published:** 2023-07-15

**Authors:** Miguel Molina-Borja, Martha L. Bohórquez-Alonso

**Affiliations:** Department Animal Biology, Facultad Ciencias, Biología, Universidad La Laguna, 38203 La Laguna, Tenerife, Spain; mlbohor@gmail.com

**Keywords:** morphology, behaviour, evolution, lizards, Canary Islands, *Gallotia*

## Abstract

**Simple Summary:**

We review the results of studies performed during the last four decades on the morphology, behaviour and evolution of lizards of the genus *Gallotia*, from the Canary Islands. We show that there is sexual dimorphism in all species of that genus, with males having larger snout-vent length (SVL), head width (HW) and hind limb lengths (HLLs) than females. The changes in SVL of males and females have been correlated throughout the evolution of these lizards in the islands. In two species, longer HLLs were found in populations from open (less vegetation) rather than closed habitats. In most species, males have a more conspicuous coloration, with blue or green large lateral spots. Blue spots in some species also have their peak reflectance in the ultraviolet part of the spectrum (300–400 nm wavelength). Detailed analysis of *Gallotia galloti* shows a large repertoire of behaviour patterns, and results of intramale competition trials show that the outcome of fights depends on several morphological patterns and especially on bite frequency. Detailed behavioural analyses of individuals of two endangered lizard species proved to be a useful tool for keeping them while in breeding centres, as well as to train them to recognize local predators before they were reintroduced into natural habitats.

**Abstract:**

We summarize, here, the results from several studies conducted over many years on several endemic species of lizards (genus *Gallotia*) from the Canary Islands. Quantitative analyses show clear differences both among the species of every island and populations within each species. Sexual dimorphism exists in all analysed species, and a phylogenetic analysis shows that the degree of dimorphism did not change along the evolutionary history of the Canary Islands: species with large and small body sizes have a similar degree of sexual dimorphism, with male body size changes closely following those undergone by females. In *G. caesaris* (from El Hierro and La Gomera islands) and in *G. stehlini* (from Gran Canaria), longer hind limb length was correlated with more open habitats. Within most species, males are more conspicuous than females, mainly in terms of body size, behaviour and coloration pattern. Lateral colour spots are blue in most species and green in others. In *G. galloti* from Tenerife, male lateral spots have larger spot areas and percentage of reflectance in the ultraviolet/blue part of the spectrum than females. This trait shows a monthly variation along April to July, both in males and females, its magnitude being larger in May–June. Behaviour analysis, especially in the last species, shows a great diversity in behaviour patterns, and analysis of intrasexual male competition revealed that contest outcome depends on several morphological and colouration characteristics but mainly on the individual’s behaviour. Detailed behavioural analyses were useful for managing a few captive individuals of the highly endangered *G. bravoana* from La Gomera island. Experimental analyses of some behaviours in the endemic Hierro island lizard (*G. simonyi*, in danger of extinction) show that individuals may learn to recognize predator models and increase their running speeds with training.

## 1. Introduction

Analysing the variation in morphology, physiology and behaviour of phylogenetically related species in relation to ecological traits is a way to understand the factors that have affected the expression of current phenotypes and, therefore, their evolution. Simultaneously, this type of analysis produces insights into the contribution of those traits to the adaptability of their bearers to the environment. This type of approach has been used for both the higher taxonomic levels (Families, Orders [1,2]) and lower levels as species and populations [3,4].

Within lizards, it has been shown in several species that the variation in morphology and behaviour is intimately associated with characteristics of the animals’ micro-habitat. Studies by Losos and colleagues [5,6,7,8] showed the inter-relationships of several lizard morphotypes and their environments in Antillean islands. This type of association has also been found in *Anolis* from Mexico and Central and South America, although island assemblages display a greater number of morphotypes than mainland assemblages [9].

Lizards of the genus *Gallotia*, endemic to the Canary Islands [10,11], are considered a basal group within Family Lacertidae [12]. There are twelve described subspecies, currently living in different islands, that fall into three size classes:

(1) Those of large body size and phylogenetically older, like *G. stehlini* from Gran Canaria island (adult males may reach a maximum snout-vent length (SVL) of 220 mm [13]; restricted populations of *G. simonyi* from El Hierro (adult males may reach 200 mm of SVL in their original natural environment [14] but may attain 250 mm of SVL in a breeding Center (Rodríguez-Domínguez et al., unpublished)); *G. intermedia* from the North-West of Tenerife [15] and *G. bravoana* from South-West of La Gomera (maximum male SVL: 199 mm, our own unpublished data). These last three species are genetically very close [15].
(2) Four subspecies of medium body sizes: *G. galloti galloti* and *G. g. eisentrauti* from Tenerife (maximum male SVL of 135 mm [16]), *G. g. palmae* from La Palma (121 mm maximum male SVL; our unpublished data) and *G. g. insulanagae* from the outer Roque de Anaga islet (maximum male SVL = 145 mm [17]).
(3) Species of small body sizes: *G. atlantica atlantica* (from Lanzarote, 96 mm male maximum SVL), *G. a. mahoratae* (from Fuerteventura, 69 mm male maximum SVL [18]) and *G. caesaris caesaris* (from El Hierro, 94 mm male maximum SVL) and *G. c. gomerae* (La Gomera, 111 mm maximum SVL of adult males) [19].

All *Gallotia* lizards are heliothermic and, in general, have an omnivorous diet [20,21]. Individuals search actively for food, and they may climb shrubs or trees to consume leaves, flowers and fruits. All species, within each island, live in practically all types of habitats, from the more xeric to the more vegetated, and even in the highest altitude of volcano Teide (3718 m.a.s.l., *G. galloti*) in Tenerife. Their densities vary among habitats and have only been evaluated partially for *G. galloti* [22,23], and in preparation, and more recently for *G. stehlini* [24]. All species are oviparous, and the number of eggs increases with female body size [25].

Lizards of the genus *Gallotia* are important for several reasons: (1) their phylogenetic position as a basal group within lacertids [12], (2) their characteristics associated with insularity, since they are one of the few totally insular lizard genera, and (3) the conservation problems they may pose (actually, three of the largest species are critically endangered) (https://www.iucnredlist.org/search?taxonomies=107634&searchType=species, accessed on 16 May 2023). Knowledge of lizard morphology, behaviour and ecology along the years has allowed us to contribute to recent attempts at recovering endangered species of *Gallotia*, known as “giant” lizards from El Hierro (*G. simonyi*) and La Gomera (*G. bravoana*). We have advised on how to maintain individuals in outdoor terraria, select the pairs to participate in reproduction every year and procedures related to antipredator training and releasing groups of lizards into the wild ([26,27] and see below).

In the current review, we summarize the main results found in different studies performed throughout the past 41 years on *Gallotia* lizards, and we also include some previously unpublished data from some species.

## 2. Materials and Methods

### 2.1. Study Sites and Species Considered

**Figure 1:**
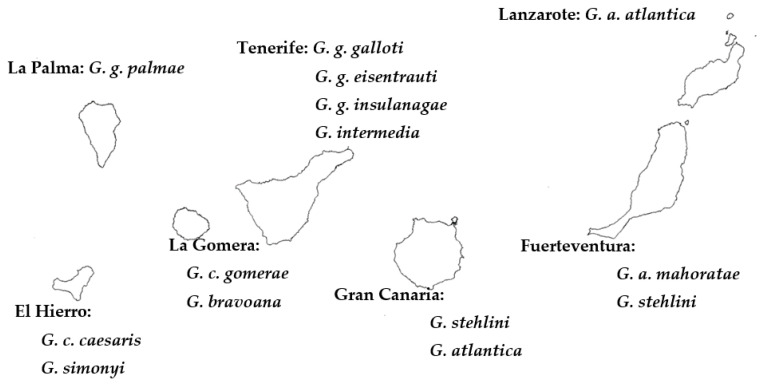
Drawing of the main larger Canary Islands, indicating *Gallotia* species and subspecies present in each one.

Since 1977, we have been sampling lizards on all of the bigger islands but not on the smallest island of La Graciosa nor in the islets of Lobos, Alegranza or Montaña Clara (Figure 1).

In Table 1, we present a list of the different *Gallotia* species studied together with the localities, islands and years when they were studied.

### 2.2. Biometric Parameters Analysed

As a standard method, in each species, we took measurements of: SVL, head width (HW), head depth (HD) and hind limb lengths (HLLs) in mm (defined as in [28]); on several occasions, we also took body mass (BM, in g), but as lizards had previously eaten part of the trap bait (or some females could be pregnant), we did not include BM in statistical analyses. HD was not considered in all comparative or sexual dimorphism analyses. For *G. galloti* (from Tenerife), we occasionally counted the number of femoral pores in males, and, in some cases, we measured the surface occupied by lateral ultraviolet-blue spots in males and females and their reflectance in the ultraviolet-visible range (300–700 nm) [16,29]. Sexual dimorphism (SD) in these traits was analysed in most *Gallotia* species. The analysis of sexual dimorphism in lizards has been commonly performed by relativizing body traits to SVL; however, in many species, the relative female trunk length has been shown to be clearly dimorphic (larger than that of males) [30,31]. Therefore, in more recent analyses of sexual dimorphism in some *Gallotia* species, we used relativized measures of body traits to trunk length (instead of SVL).

Some of the morphological traits measured are important in lizard behaviour. For example, head size has an influence on the outcome of intramale competition [32,33,34]. On the other hand, size of hind limbs is known to affect running speed and the capacity to climb [35,36].

### 2.3. Life History Traits

Assuming a monophyletic origin of all the species of *Gallotia*, as they are closely related based on genetic distance data [37], a comparative analysis of biometric, life history and other data may provide useful information on the evolutionary relationships among them. Pioneer studies in this sense were the works of Thorpe and collaborators in the 1980s and 1990s [38,39].

**Figure 2:**
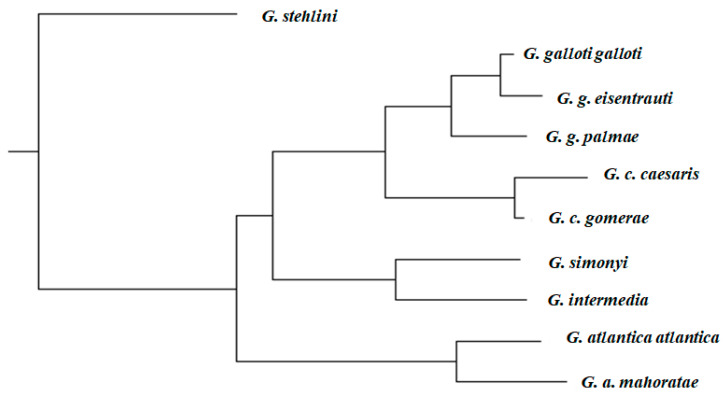
Phylogenetic tree of *Gallotia* species used for comparative analyses of biometric and life history traits (taken with permission from Journal of Zoological and Evolutionary Research–JZER-[25]).

For this type of analysis, we used data compiled from ten species/subspecies of *Gallotia* (the only species for which there were genetic sequences at that time [37], Figure 2) and used Felsenstein’s method of phylogenetic comparative analysis [40]. The aim was to perform an analysis of sexual dimorphism, SVL at sexual maturity and life history data as the number of eggs laid by females [26]. We also analysed the relationships between SVL and other biometric traits within each sex and the association between female SVL and life history data (SVL and age at maturity, adult life span and hatchling length and mass). These analyses had the additional aim of revealing if Canarian lizards followed the same or a different evolutionary pattern of life history traits than continental (European) lizards [4,25].

### 2.4. Behavioural Patterns

In the initial phase of our research, we analysed, in detail, and described the repertoire of natural, spontaneous behavioural patterns (ethogram) expressed by individuals of *G. galloti* [41,42] and some occasional observations of eating in *G. stehlini* [43]. We then described courtship and mating behaviour of *G. simonyi* [44] and also made behavioural observations for six individuals of the critically endangered *G. bravoana* initially included in the first attempts of reproduction in captivity [45]. For all these studies, we used the classic methodology in ethological research. We initially described their behavioural patterns and a posterior quantification of their frequencies or durations [41,42,43,44,45,46,47].

In the specific case of analysing the aggressive behaviours within males, we used an experimental protocol that consisted of establishing pairs of individuals (randomly chosen between those available in the laboratory that were previously captured in several field locations) that were put in a neutral terrarium. There, they were allowed to interact for a maximum of 30 min, and their behaviours were filmed. A posterior detailed analysis of the recorded videos allowed us to quantify the frequency (or duration) of the several behaviour patterns performed by each contestant and analyse factors affecting contest outcome [33,34].

### 2.5. Application to Actions for Endemic Lizard Conservation

During the last few decades, we have collaborated with the Cabildos (i.e., town councils) of El Hierro and La Gomera to provide advice on the maintenance and reproduction of individuals (*G. simonyi* and *G. bravoana*, respectively) in each breeding centre of the islands and also in the reintroduction of individuals into the wild. In preparation for release into the wild, we have trained individuals of *G. simonyi* to recognize dangerous predators (kestrels and cats) using models [26], in order to increase their chances of surviving real attacks when they are reintroduced in natural habitats; we also showed that experimental trials contributed to an increase in their running speed [27].

### 2.6. Statistical Analyses

In all studies performed, we used several statistical analyses, from the simpler, nonparametric comparisons between two or more samples [29,31,43], ANOVA or MANOVA [16,18,19,48], to the more complex, multivariate (principal component analysis) [29], phylogenetic analysis [25], permutational analysis of variance (PERMANOVA) [26] and generalized linear models [27,32].

### 2.7. Ethical Considerations

In all cases, when lizards were manipulated in the field or participated in lab experiments, we followed the guidelines published by Animal Behaviour (ASAB/ABS 2012; Anim. Behav. 83: 301–309). Lab experiments received official approval from the Ethics and Animal Welfare Committee of the Universidad La Laguna (reference CEIBA2011-0020).

## 3. Results and Discussion

### 3.1. Sexual Dimorphism and Body Traits

Sexual dimorphism (SD) is present in every *Gallotia* species, with males having larger bodies and, in some species, being more colourful than females. In Table 2, we present descriptive statistics for SVL, HW and HLL analysed in both sexes of several species and populations within the main islands. Mean values for the three traits are clearly larger in males than in females and they were significantly different [18,19,28].

Patterns of SD in lizard body size (SBSD) include those with males having larger SVL than females, females larger than males and no sexual dimorphism in SVL [49,50]. The resulting SBSD pattern depends on evolutionary and ontogenetic factors that may have affected males and females differentially; for example, larger trunk relative to SVL may be better explained in relation to female size (fecundity hypothesis), both in some lacertids [31] and in many other lizards [51,52], and sexual selection for large male size (intrasexual male competition) may explain SBSD in species with larger males [53,54]. These have been the main hypotheses to explain SBSD in lizards. However, a comparative phylogenetic analysis of 497 lizard populations of 302 species showed that territoriality and clutch size were significant predictors of SBSD, but only 16% of the variation was explained using these variables [52]. Therefore, the authors suggested that alternative additional hypotheses and proximal mechanisms should be considered. On the other hand, by analysing growth plate cartilage resorption, it has been recently shown [55] that many squamates (including *G. galloti* and *G. stehlini*) exhibit a determinate growth (they do not continue growing after sexual maturity), which would limit them to reach large body sizes. However, our experience measuring SVL in *G. simonyi* and *G. bravoana* shows (unpublished results) that individuals continue growing after sexual maturity, which would agree with the finding that monitor lizards show an indeterminate body growth [56]. In the case of *G. galloti*, measurements in at least two recognized adult individuals in two different years showed an increase in SVL (our own unpublished results). This does not necessarily contradict the results of Frýdlová et al. [55], as individuals showing determinate growth can still continue growing after sexual maturity, and/or indeterminate growers may exhibit asymptotic growth [57].

Sexual dimorphism is also present in lizard head size [58], with males usually having relatively larger heads than females [59]. This finding has been interpreted as resulting from intra-male competition favouring larger heads that would increase the probability of winning aggressive contests [33,34,53] (see Behaviour section, below). We have shown that sexual dimorphism in head size (width) is also present when we relativized this trait to trunk length and not to SVL [13]. In *G. galloti*, SD in head size has also been related to the male to female niche divergence hypothesis [59]. SD in head size has also been analysed considering its relation to other ecological characteristics; thus, for example, island-specific effects (environmental features of islands) affected head shape variation (and SD) in *Podarcis muralis* populations from several islands of the Tuscan archipelago [60].

**Figure 3:**
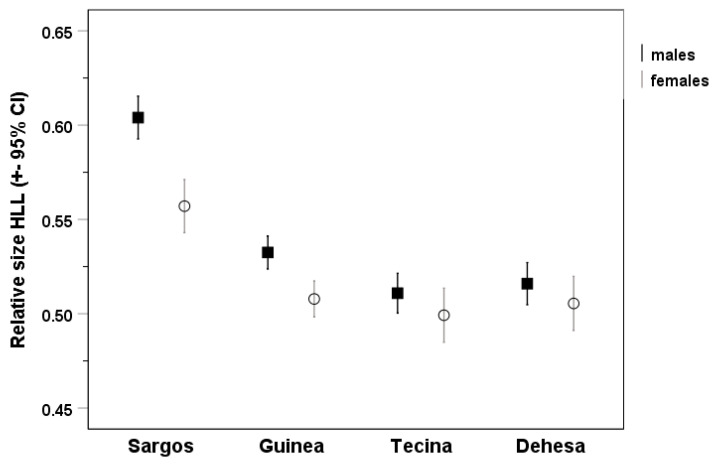
Relative HLL for males and females of four populations of *G. caesaris.* Figure redrawn from that published in *Journal of Herpetology* [19].

For HLL, sexual dimorphism also exists in many lizard species. Typically, males have longer limbs than females [61,62]. This trait has also been related to ecological characteristics; in the specific case of *G. caesaris*, we showed that HLL was related to the type of habitat, with longer hindlegs in open than in closed habitats (Figure 3 [19]). This is a phenomenon found in several lizard species [63,64], including *G. stehlini* [13], and may also be present in other *Gallotia* species.

**Figure 4:**
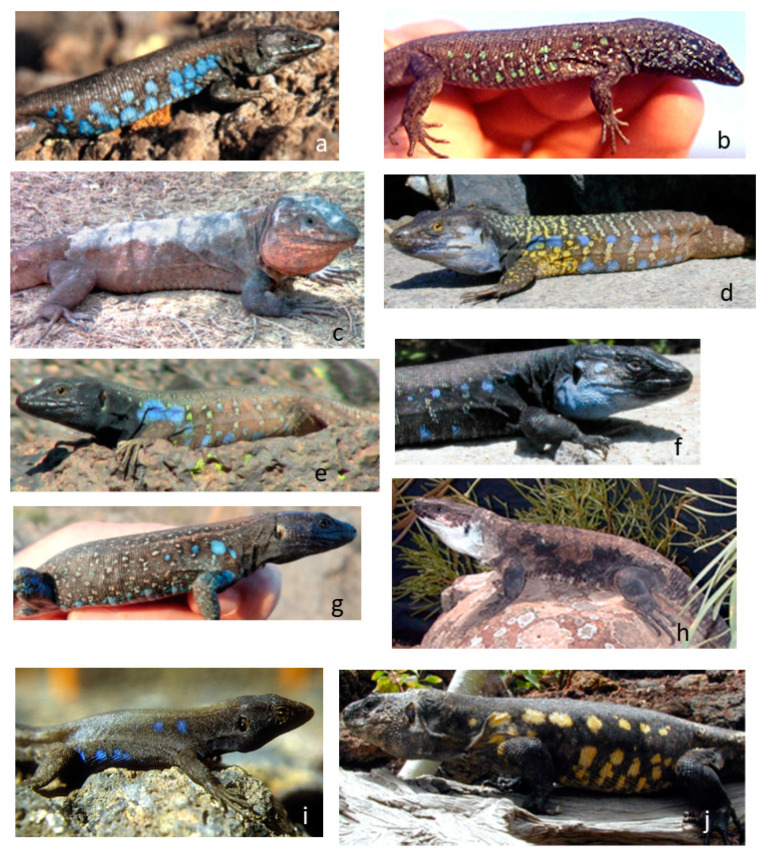
Pictures of males from different *Gallotia* species, showing the presence (or not) of coloured lateral spots. (**a**) *G. a. atlantica*, (**b**) *G. a. mahoratae*, (**c**) *G. stehlini*, (**d**) *G. g. eisentrauti*, (**e**) *G. g. galloti*, (**f**) *G. g. palmae*, (**g**) *G. c. gomerae*, (**h**) *G. bravoana*, (**i**) *G. c. caesaris*, (**j**) *G. simonyi*. Sizes of pictures not scaled to lizard sizes (see Table 2 for mean male and female adult size of each species).

**Figure 5:**
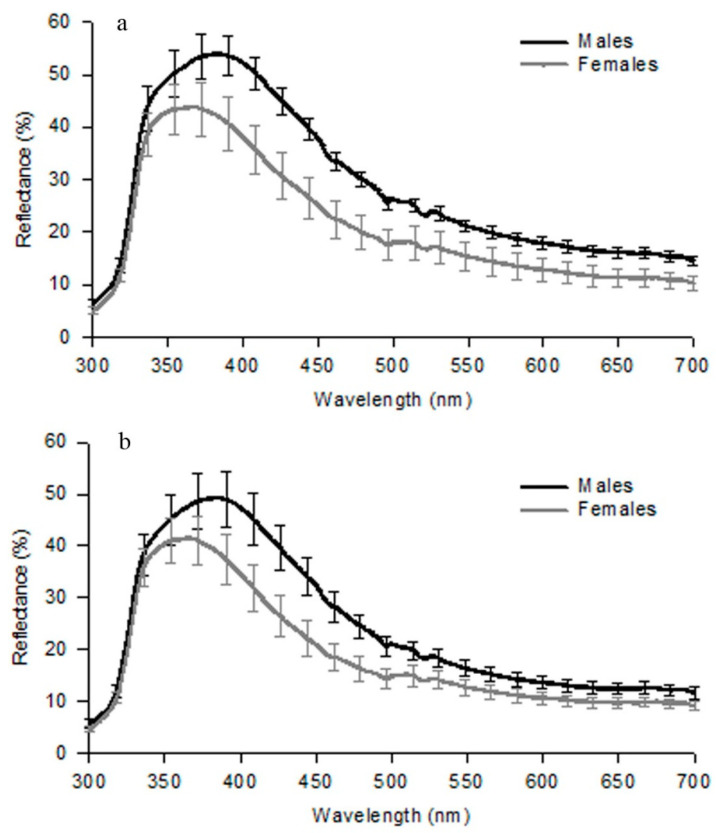
Mean reflectance (±S.E.) from first lateral UV-blue spot (300–700 nm wavelength) of males and females *G. g. galloti* (**a**) and *G. g. eisentrauti* (**b**) (taken with permission from *Journal of Zoology* [29]).

In Table 3, we present a summary of the colours as they appear to the human eye of male lateral spots in the *Gallotia* species. Except for some populations of *G. atlantica* that have green lateral spots, *G. simonyi* with yellow to orange spots and *G. stehlini* with no lateral spots, all other species have blue lateral spots (Figure 4). In *G. galloti*, which we analysed in detail, male lateral spots are blue in the visible range, but their peak reflectance is below 300 nm, in the UV range of wavelengths (Figure 5a,b [29]). Reflectance in the UV is important because most lizards are capable of perceiving these wavelengths [65]. UV-blue spots have been described in other lacertid species (*Podarcis*), where they play a role as signals of fighting ability [66].

We also showed that the reflectance spectra (300 to 700 nm) from these lateral spots changed seasonally, luminance being more developed (both in males and females) during May and June (breeding time) than in April or July [16]. The magnitude of the reflectance from these lateral spots, particularly in the UV range, was found to be larger than that of other lacertid species from the Iberian Peninsula [67,68].

Moreover, male lateral coloration partly influenced the outcome of male intrasexual contests of *G. galloti* [33,34] (see below). Secondary sexual colouration often reflects the condition of the individuals, and it has been shown in *G. g. palmae* that the expression of cheek patch colour is related to the load of parasites [69].

### 3.2. Evolution of Morphology and Life History Traits

**Figure 6:**
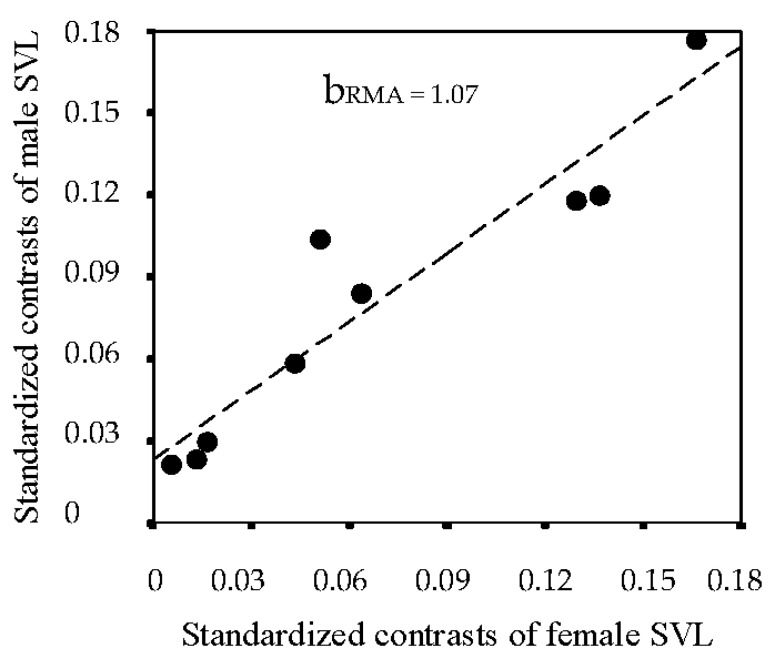
The relationship between standardized contrasts of male SVL (*Y* axis) on female SVL (*X* axis). Redrawn from JZER [25].

The phylogenetic analysis of ten species of *Gallotia* showed that independent contrasts of SVL in males were significantly correlated with those of females, the slope of the regression line not significantly different from 1.0 (see Figure 6). This suggested that changes in the body size of females and males have been correlated throughout evolution [25].

The evolution of large body size of lizards on islands has been associated with increased resources on larger islands [70]. This could also happen along the colonization progress of lizards on the Canary Islands. The largest species were living or are still present in the more western and more vegetated islands (Gran Canaria, Tenerife, La Palma, La Gomera and El Hierro). Taking into account that *G. atlantica* lives in the oldest Fuerteventura Island (around 20 million years old) and *G. caesaris* in the youngest El Hierro island (around one million years old), small lizard body sizes could have evolved twice along the rise of the Canary Islands. Lacertids from the European continent (for example: *Psammodromus*, *Algyroides*, *Lacerta* or *Podarcis*) are commonly smaller than some *Gallotia*, except for *G. caesaris* and *G. atlantica* on the islands and *Timon lepidus* on the continent [71]. It had been suggested that this could have been the pattern of lizard colonization in these islands from the oldest and most eastern Fuerteventura to the newest and most western of El Hierro [72]. The discovery of fossils from a large lacertid in mainland Germany living by early Miocene shows that large lizards were already present on the continent before the emergence of the oldest Canary Islands [73] and could have been potentially at least one of the first lizard colonizers. However, as no fossil of a large *Gallotia* has been found up to now on the eastern islands of Fuerteventura and Lanzarote, the first large lizard to evolve (*G. stehlini*, probably from a species coming from those eastern islands) was in Gran Canaria [72] and, later on, on the more western islands (*G. simonyi*, *G. intermedia*, *G. bravoana* and the extinct *G. auritae*). Evolution among different islands of lizard body size and other morphological, physiological and ethological traits may be due to several factors, including genetics and ecological conditions (food resources and predators) [74]. For example, environmental conditions had to affect lizard evolution in the islands; the colonizers of the first islands arising from the sea bottom probably would have faced harsh conditions due to the volcanic substratum with arid conditions, at least initially (lizard colonization could have occurred long after island emergence [72]). There is no suggestion of what the first plants to colonize the emerging islands were; however, a research team deduced that in the last few millions of years, many herbaceous plants changed to woody shrubs or trees in the Canaries [75]. Potentially, reduced feeding sources as herbs, small shrubs and insects would have implied reduced water availability for the initial lizard colonizers, and experimental studies have shown that dehydration reduces lizard preferred body temperature. This may lead to non-effective thermoregulation and, therefore, affect fitness and life history traits [76]; however, lizards are capable of compensating for reduced water intake [77], which could have helped the first island colonizers facing harsh conditions in emerged islands.

**Figure 7:**
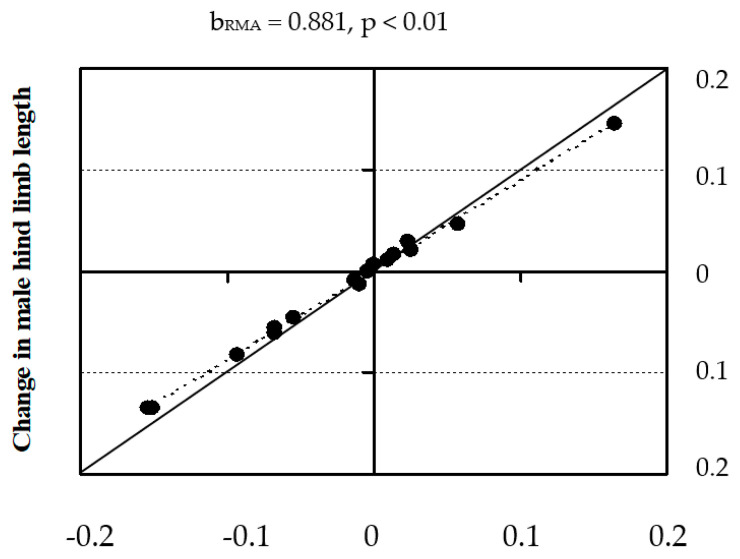
Graph of the inferred changes in male SVL and male hind-limb lengths. Points represent the changes occurring along each of the branch segments in the phylogenetic tree. Dashed line is the regression line adjusted to the points. Continuous line, at 45° angle, represents perfect coadaptation (Taken from JZER [25]).

The evolution of head and body traits was always correlated to changes in SVL (regression slopes not significantly different from 1) within each sex (see details in Table 4 [25]). However, in the particular case of males, hind limb lengths changed proportionally less than SVL (negative allometry) (Figure 7 [25]); that is, those of larger species have comparatively shorter limbs than those of smaller species. Though we suggested that larger males in the largest species could have some disadvantage in relation to running very fast to escape from predators, shorter limbs also imply a reduced force needed to keep the joint at equilibrium [61].

The number of eggs laid by females was significantly related to their snout-vent length (regression slope significantly higher than expected value, see Table 4) [25]. In general, the correlated evolution of life history traits and female SVL reflects that body size is a major factor affecting these traits. This is the rule in many vertebrate and invertebrate species [78].

It has been hypothesized that insular lizards should show what is known as insular syndrome, mainly characterized by females producing few but larger offspring than the species on the mainland; this was confirmed considering a huge list of lizards, including some *Gallotia* species [79]. In the case of lizards from this genus, considering *Psammodromus*, the closest extant genus to *Gallotia* and *P. algirus* (maximum female SVL = 74 mm [80]) in comparison with *G. caesaris* (with a similar female SVL range: 76–83 mm, Table 2), mean clutch size is larger in the first species, but hatchlings are larger in the second [25,80]. Moreover, in our comparative analysis, as hatchling SVL increased less than female SVL (regression slope significantly less than 1 [25]), it means that hatchling lengths are comparatively smaller in the larger than in the smaller species. Correspondingly, smaller species have a relative clutch size smaller than that of larger species [25], our unpublished calculations. These data, together with the impact of predators (including humans), could help explain the fate of the larger *Gallotia* species (with “slow” life history traits [78]), three of which are currently in danger of extinction.

It was suggested by Novosolov and Meiri that [79]: (1) reduced clutch sizes could have resulted from limited resources on the islands, but, with time, endemic species could have become better adapted to the insular environment and increased their clutch size. This could have been the case if a small *Gallotia* species was the first to colonize the first emerged island and, later on, larger species (with an increased relative clutch size) did evolve. However, the scenario is not clear yet, as a small species (*G. atlantica*) developed on the first island to emerge (Fuerteventura), and larger species (*G. stehlini* and *G. simonyi* group) developed, respectively, in Gran Canaria and El Hierro islands, the second and the last to emerge [72]; (2) few but large offspring are due to few predators on oceanic islands (the reverse happening on continental islands with many predators). The latter could have happened on islands where large *Gallotia* developed (the most western islands, more separated from the continent); however, as we said above, offspring from larger species are comparatively smaller than in the smaller species.

### 3.3. Activity Cycles

**Figure 8:**
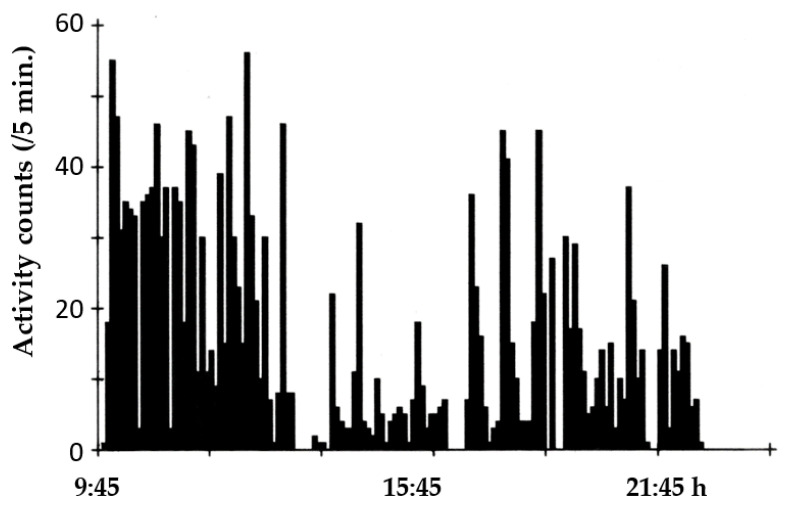
Variation in locomotor activity of an individual of *G. galloti* recorded during 12 h under controlled light–dark (12:12 h) and temperature daily cycle.

All species of *Gallotia* are diurnal (but see [81]), and their seasonal change is characterized by having greater daily activity on longer and warmer days of spring and summer [48,82]. However, some lizard activity is also evident in warmer days of winter and autumn. Newborns are especially active on warmer days of autumn (our unpublished observations). Daily cycles in the field have not been studied, but experiments performed in lab terraria with controlled light–dark and temperature cycles showed that individuals have a main active period during the morning, which is reduced at midday and partially recovered in the afternoon *(*Figure 8) [83].

### 3.4. Behaviour Patterns

**Figure 9:**
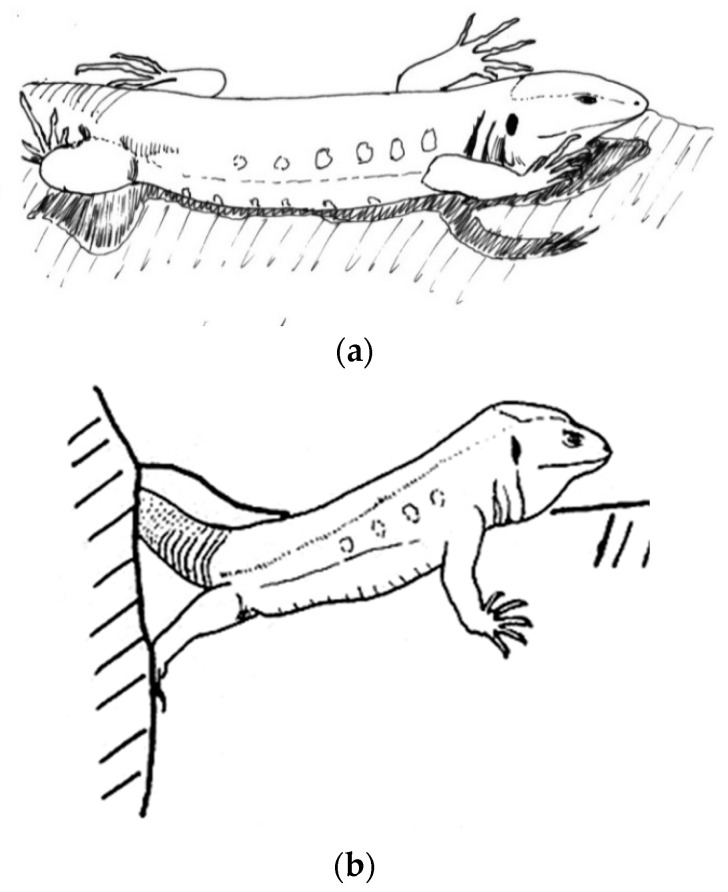
Drawings (taken from pictures) of two behaviour patterns of *G. galloti*: (**a**) four legs raised from the substratum; (**b**) gular extension in an adult male in the initial phase of agonistic contest; (**b**) taken with permission from Revista Española de Herpetología [87].

Behaviour patterns in different species of *Gallotia* are very similar, though no systematic research has been performed up to now in all of them. However, they differ, for example, in the sequence of head bobs performed by males during courtship. For example, we described nine head bobs (of large and small amplitude) occurring in 5 s for a male courtship sequence in *G. g. eisentrauti* [84], but the amplitude and duration of head bobs seem to be slower in larger species, such as *G. intermedia* (unpublished data) and *G. bravoana* [45]. In *G. galloti*, the whole repertoire of behaviour patterns included more than 40 different behaviour patterns [41,42,46,47]. In Figure 9, we include two examples: drawings of a typical behaviour pattern shown by a male of *G. galloti* raising all four legs when the substratum is very hot (a), and the posture extending the throat of a male while in the first stage of agonistic and courtship sequences (b). On the other hand, it is characteristic of *Gallotia* that males bite the female neck during mating [85], which differs from other lacertids (like *T. lepidus*) in which the male bites the female in the trunk.

The analysis of male intrasexual contests in *G. galloti* led to the discovery that body and head size, frequency of aggressive patterns (particularly bite frequency [34]) and reflectance of ultraviolet/blue lateral patches influenced the outcome of the contests, being larger or higher in winners than in losers [33,34]. The results of these experiments led us to conclude that though several morphological, including colouration, traits affect the probability of winning a contest, behavioural traits (specifically bite frequency) were the best predictor of the contest outcome [34,86].

Though the same type of detailed analysis has not been performed up to now on other *Gallotia* species [87], there are some particularities in some behaviour patterns expressed, for example, during aggressive interactions in *G. stehlini* (from Gran Canaria Island). Occasional observations of male intrasexual encounters in lab experiments showed that agonistic interaction could be particularly intense in this species. Males could remain with interlocked jaws for more than 10 min (our own unpublished data).

### 3.5. Space Use

**Figure 10:**
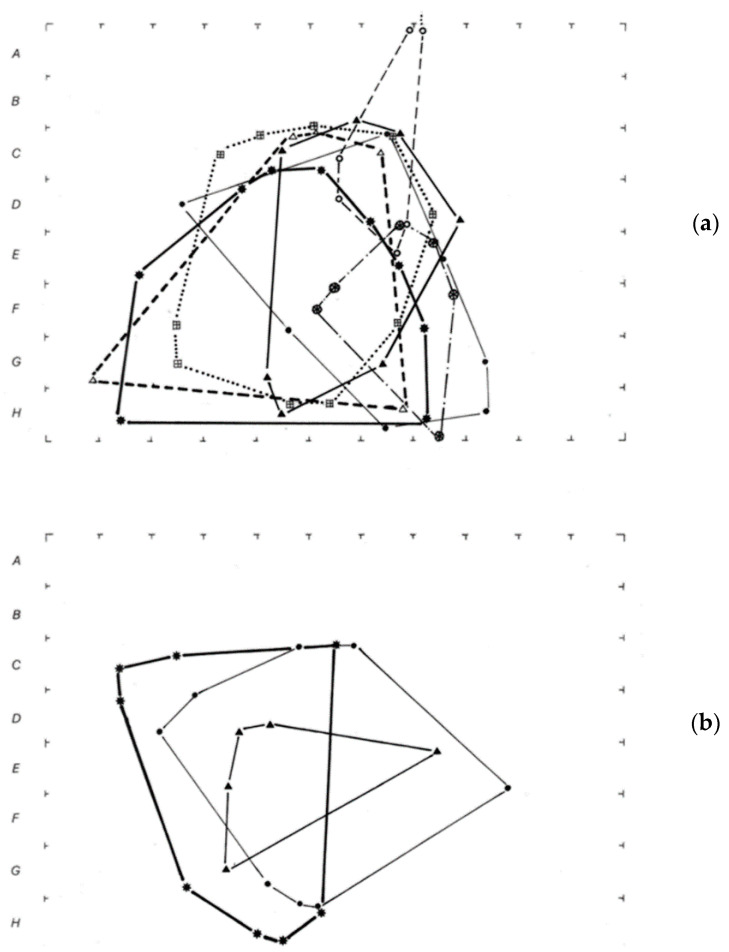
Home range areas calculated from direct observations of some individual adult males (**a**) and females (**b**) from a NW locality of Tenerife; different line types correspond to individual lizards. Crosses close to letters indicate every two-meter mark placed in the ground as a reference to calculate home areas (taken with permission from Bonner zoologische Beitrage [88]).

Based on direct observations of lizards (*G. galloti*) individually recognized by their natural marks, the maximum calculated home areas for several male and female individuals were 89.2 and 77.7 m^2^, respectively (Figure 10 [88]). Another study [22], based on a grid of capture traps in a south-east population, estimated a home area of around 100 m^2^ in several habitat types of the area. No other published home range estimation exists for any other *Gallotia*, except for the calculation of 200–300 m^2^ of two *G. simonyi* individuals that were experimentally released into the wild provided with a transmitter [89].

Several types of territoriality have been described [90], and based on our results, in the case of *G. galloti,* given the great overlapping of home area within males, within females and between both sexes [88], no strict territoriality is supported. The typical displays and combats between males correspond to type IV of the defence area described by Martins (Table 6.1 in [90]), which includes the occurrence of combat and defence by males of specific sites but not of the whole individual’s area. For a review of territorial (or non-territorial) lizard species of several families, see [91].

### 3.6. Body Orientation

**Figure 11:**
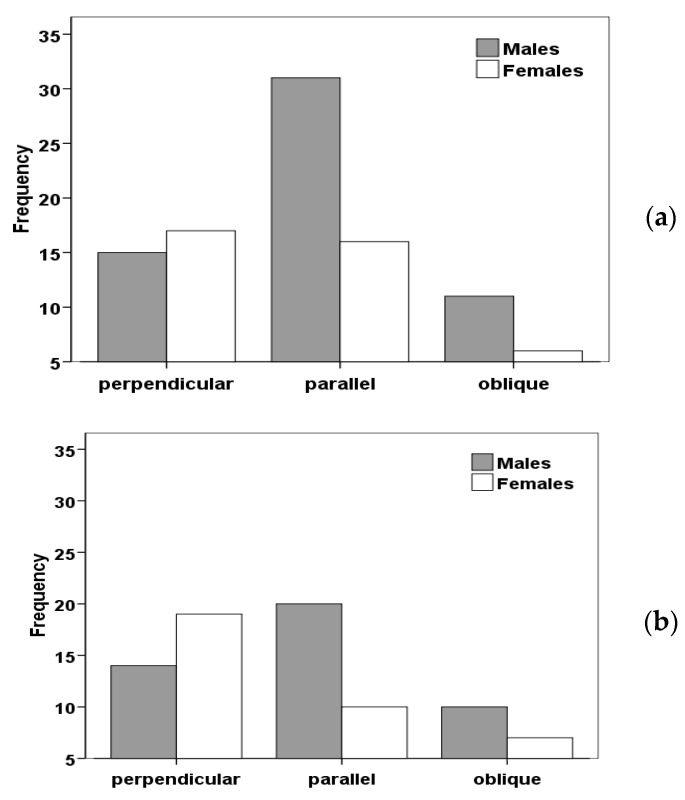
Frequency of three body positions in males and females of *G. g. galloti* in Güimar (SE Tenerife) during the morning (**a**) and midday (**b**) daily periods (taken with permission from Amphibia-Reptilia [48]).

After years of behavioural observations from lizards in different localities of Tenerife, we asked ourselves if they could adopt particular body positions in relation to the sun rays in different parts of the day. Therefore, by means of detailed observations performed along transects in the field, we quantified three main body positions of lizards (*G. galloti*) in relation to sun and in two daily hour periods [48]. The results showed that male body position parallel to sun rays was more frequently shown than perpendicular or oblique in two localities of Tenerife (Figure 11 [48]); this was interpreted taking into account several benefits and costs of adopting different body orientation in relation to environmental (biological: conspecifics) and physical factors (temperature). Thus, parallel orientation minimizes the body surface that is displayed to the sun, thus reducing the absorbance of radiant heat during the hottest part of the day. However, as operative temperatures were not significantly different in metal tubes experimentally set up in parallel or perpendicular orientation, this factor does not seem to be functional in relation to body temperature regulation by lizards. An alternative hypothesis is that, with parallel body orientation, lizards would have similar light intensity and spectral characteristics reaching both eyes (located laterally in their heads); this could help in obtaining important visual information on conspecifics or predators [48]. With parallel body orientation, lizards would also have both sides of the trunk equally illuminated, at least during midday hours, thus being conspicuous to conspecifics located to their left and to their right. As detailed above, blue patches of *G. galloti* are located on the sides of the body, and we showed that they are an important factor affecting male fighting ability [34]. Being more conspicuous can be disadvantageous in relation to predators, but lateral coloration should be almost undetectable, as seen from above by kestrels or buzzards, the main lizard predator (up to now, there is no snake in natural habitats of Tenerife (see [48] for a full discussion of those factors)).

### 3.7. Behavioural Applications on Conservation of Endemic Lizards in Danger of Extinction

We have been advising Breeding Centres of *G. simonyi* and *G. bravoana* for many years, selecting male and female individuals (since at least 2000, *G. simonyi* and since 2009, *G. bravoana*) to participate in each year’s reproduction (in preparation). Experiments on *G. simonyi* showed that individuals could be trained to recognize main predators as kestrels and cats using stuffed specimens [26]. This was achieved by using a standard learning protocol, including pre- during and post-training periods, during which lizards’ behaviours were recorded. The statistical comparisons showed that most of the individuals reduced their activity outside burrows or directly fled during or after the stimulus presentation. We recommended using this training technique for lizards to be reintroduced into natural habitats. Experiments on locomotion training for individuals of the same species showed that they enhanced individuals’ running speed [27].

## 4. Conclusions

Considering all the above results from our research over the years, we concluded that lizards of the genus *Gallotia* show: (1) Specific particularities in body size and colouration in relation to other lacertids. The evolution body traits in lizards from these islands have been mainly characterized by the presence of both species with small and large body sizes at different geological ages of island emergence. (2) On the other hand, they show general trends in body parameters as head size and hind limb size (larger in males than in females) common in many other lizards, even outside the Lacertidae family. At least two *Gallotia* show larger HLL in open rather than in closed habitats, consistent with findings for other lizard species.

Colouration patterns, specifically in males of *G. galloti* from Tenerife and La Palma and those from some populations of *G. atlantica* (Lanzarote and Fuerteventura), are particularly conspicuous (highly contrasting blue or green lateral spots) in comparison to other species of the genus (like *G. bravoana* and *G. stehlini*) and other lacertids (except, for example, *P. algirus* or *T. lepidus*). Moreover, the spectra of those lateral spots have a larger component in the ultraviolet range (300–400 nm) than that found in other lacertids. This coloration pattern had some influence during male intrasexual contests, but its functional significance in relation to habitat characteristics remains to be elucidated.

The behaviour patterns of *Gallotia* show some convergence with those of many other lizard species, especially in male courtship patterns (gular inflation, body flattening and head bobs). Female neck biting by males during mating is similar to what happens in other lizard families but differs from that of other lacertids, in which males bite the female trunk skin.

Individuals of endangered *G. simonyi* could be trained to recognize models of local predators.

## Figures and Tables

**Table 1:** Species, localities, altitude and year of several *Gallotia* species studied along the past 41 years. m.a.s.l.: meters above sea level.

Species	Locality (Island)	m.a.s.l.	Years
*G. a. atlantica*	Punta Mujeres (Lanzarote)	60	1998, 1999
*G. a. mahoratae*	Malpaís Arena (Fuerteventura)	178	1998, 1999
*G. bravoana* ^(^*^)^	Breeding Center (La Gomera)	34	2022
*G. c. caesaris*	Los Sargos (El Hierro)	86	1999–2001
	Guinea	82	
	La Dehesa	370	
*G. c. gomerae*	Tecina (La Gomera)	62	1999–2001
	Valle Gran Rey	26	2021
*G. g. eisentrauti*			
	Pris	82	2004–2006
	Pris	82	2008
	Pris	82	2009
*G. g. galloti*	Abrigos del Poris	14	1981
	M.P. Güimar	12	2008
	El Portillo (Teide N.P.)	2120	1996–1999, 2012
*G. g. palmae*	Tazacorte (La Palma)	205	2000–2004
	Fuencaliente	320	2000–2004
*G. simonyi* ^(^*^)^	Lagartario Guinea (El Hierro)	82	2018, 2022
*G. stehlini*	Gáldar (Gran Canaria)	50	1996, 1998–1999
	Tasartico	175	1996, 1998–1999

^(^*^)^: Morphological data from these species have not been published yet.

**Table 2:** Descriptive statistics for snout-vent length (SVL), head width (HW) and hind limb length (HLL, all in mm) of species and populations of *Gallotia* sampled in Canary Islands.

Island/Site							
(*Species*)	Sex	Trait	Mean	S.E.	Min.	Max.	N
Tenerife–Teide (*G. g. galloti*)	m	SVL	107.41	0.84	82.00	122.00	96
	f		87.32	0.67	70.00	108.00	98
	m	HW	15.89	0.45	10.10	23.44	71
	f		11.92	0.32	8.46	17.06	59
	m	HLL	57.06	0.71	41.60	66.22	50
	f		46.89	0.61	40.88	60.10	53
Tenerife–M.P. Güimar-(*G. g. galloti*)	m	SVL	112.32	1.36	80.00	126.00	59
	f		91.36	1.26	77.00	110.00	39
	m	HW	19.71	0.23	14.44	22.04	59
	f		14.64	0.16	12.60	16.66	39
	m	HLL	60.89	0.58	48.34	69.53	58
	f		48.83	0.75	38.57	55.98	39
Tenerife–Pris-(*G. g. eisentrauti*)	m	SVL	120.62	1.21	100.0	135.0	52
	f		94.41	1.43	75.00	106.0	37
	m	HW	20.23	0.22	15.63	23.75	51
	f		14.92	0.20	12.26	17.20	37
	m	HLL	63.96	0.81	50.40	74.62	51
	f		48.69	0.64	38.92	55.62	37
La Palma–Fuencaliente-(*G. g. palmae*)	m	SVL	96.70	1.23	70.82	109.57	56
	f		82.78	0.86	60.30	100.36	84
	m	HW	11.74	0.22	8.56	16.11	56
	f		9.59	0.10	7.90	12.83	84
	m	HLL	50.94	1.24	32.0	65.0	56
	f		43.60	0.79	29.0	64.0	81
La Palma–Tazacorte-(*G. g. palmae*)	m	SVL	102.95	0.68	82.44	121.16	161
	f		88.44	0.67	69.75	111.61	117
	m	HW	12.48	0.11	8.84	15.80	162
	f		10.94	0.81	7.62	105.0	117
	m	HLL	53.91	0.68	32.0	69.0	161
	f		46.75	0.62	30.0	63.0	117
El Hierro–Los Sargos-(*G. c. caesaris*)	m	SVL	75.32	1.04	62.00	84.0	31
	f		69.61	0.72	62.00	76.0	33
	m	HW	8.48	0.09	7.14	9.19	30
	f		7.73	0.08	6.92	8.75	33
	m	HLL	44.94	0.56	38.15	50.35	31
	f		38.66	0.34	34.58	42.24	33
El Hierro–Guinea (*G. c. caesaris*)	m	SVL	72.50	0.93	58.00	87.0	52
	f		67.61	0.68	57.00	77.0	49
	m	HW	8.75	0.12	7.02	10.43	52
	f		7.76	0.08	6.62	8.81	49
	m	HLL	38.29	0.47	32.91	46.50	51
	f		34.25	0.32	28.80	38.58	49
El Hierro–Dehesa-(*G. c. caesaris*)	m	SVL	77.28	1.22	65.00	94.0	36
	f		72.26	1.14	63.00	83.0	27
	m	HW	8.79	0.18	6.52	11.02	38
	f		7.82	0.11	6.10	8.66	27
	m	HLL	39.29	0.93	28.04	49.02	35
	f		36.38	0.44	31.55	41.99	27
La Gomera–Tecina (*G. c. gomerae*)	m	SVL	96.35	1.98	77.00	111.0	23
	f		82.60	0.93	69.00	92.00	30
	m	HW	11.61	0.30	8.97	13.59	23
	f		8.96	0.11	7.26	10.13	30
	m	HLL	48.89	1.05	39.77	57.02	23
	f		41.08	0.32	36.62	43.68	30
El Hierro–Breeding Center-(*G. simonyi*)	m	SVL	198.61	3.85	144	226.0	31
	f		182.04	2.5	143	204.0	25
	m	HW	19.98	0.41	15.10	25.10	31
	f		17.45	0.29	14.60	20.10	25
	m	HLL	97.70	1.41	79.30	114.20	31
	f		88.30	0.75	76.7	96.10	25
Lanzarote–Punta Mujeres-(*G. a. atlantica*)	m	SVL	85.92	1.95	59.0	96.0	25
	f		64.88	0.83	57.0	73.0	21
	m	HW	10.02	0.23	7.03	11.90	25
	f		6.99	0.09	6.27	7.71	21
	m	HLL	45.78	0.89	34.2	52.60	25
	f		30.97	0.51	24.34	35.38	21
Fuerteventura–M.P. Arena-(*G. a. atlantica*)	m	SVL	62.76	0.94	53	69.0	26
	f		56.35	0.71	45	61.0	27
	m	HW	7.41	0.13	6.08	8.54	26
	f		6.16	0.07	5.6	6.85	27
	m	HLL	33.54	0.42	29.08	36.78	26
	f		27.39	0.35	22.67	30.73	27
Gran Canaria–Gáldar-(*G. stehlini*)	m	SVL	146.15	6.49	82.0	220.0	34
	f		140.35	3.6	105	170.0	26
	m	HW	15.98	0.81	9.58	26.92	33
	f		14.7	0.43	11.06	18.38	26
	m	HLL	71.08	2.9	43.4	98.13	33
	f		65.47	1.45	52.91	86.55	26
Gran Canaria–Tasartico-(*G. stehlini*)	m	SVL	146.84	6.81	88.0	220.0	25
	f		137.52	4.23	100.0	180.0	23
	m	HW	17.66	1.02	10.85	30.10	24
	f		16.31	0.64	11.5	22.80	23
	m	HLL	77.95	2.84	52.5	110.0	24
	f		73.68	2.13	56.2	100.0	23

**Table 3:** Species, localities and main colouration in the human visible range of lateral spots from several *Gallotia* species.

Species	Locality (Island)	Colouration
*G. a. atlantica*	Punta Mujeres (Lanzarote)	blue
*G. a. mahoratae*	Malpaís Arena (Fuerteventura)	green
*G. bravoana*	Breeding Center (La Gomera)	small blue spots
*G. c. caesaris*	Los Sargos (El Hierro)	blue
	Guinea	blue
	La Dehesa	blue
*G. c. gomerae*	Tecina (La Gomera)	blue
	Valle Gran Rey	blue
*G. g. eisentrauti*	El Pris (N Tenerife)	blue
*G. g. galloti*		
	M.P. Güimar (SE Tenerife)	blue
	El Portillo (Teide National Park)	blue
*G. g. palmae*	Tazacorte (La Palma)	blue
	Fuencaliente	blue
*G. simonyi*	Guinea Breeding Center	
	(El Hierro)	yellow-orange
*G. stehlini*	Gáldar	
	(N Gran Canaria)	no lateral spots
	Tasartico (SW)	male gular skin yellowish
		to light brown

**Table 4:** Summary statistics of relationships between head and body traits and mean adult male length (**a**) and mean adult female length –or mass- (**b**) using Felsenstein’s independent contrasts calculations (FL1P) and “minimum evolution” method (ME1P). Significance tests for ME1P are based on empirical null distributions created through computer simulations; those for FL1P based on conventional critical values (from [25]). n.s.: non-significant.

**(a)**							
Dependent variable		r	P*_r_*	b_exp_	b_OLS_	b_RMA_	P_RMA_
Mean Head Width	FL1P	0.984	***	1	0.898	0.913	n.s.
	ME1P	0.988	**	1	0.894	0.904	n.s.
Mean Head Depth	FL1P	0.974	*	1	1.039	1.068	n.s.
	ME1P	0.976	**	1	1.061	1.086	n.s.
Mean Fore Limb Length	FL1P	0.996	***	1	0.998	1.001	n.s.
	ME1P	0.991	**	1	0.995	0.998	n.s.
Mean Hind Limb Length	FL1P	0.998	**	1	0.880	0.881	**
	ME1P	0.998	**	1	0.885	0.886	**
(**b**)							
Dependent variable		*r*	P*_r_*	b_exp_	b_OLS_	b_RMA_	P_RMA_
Mean Male SVL	FL1P	0.973	**	1	1.042	1.070	n.s.
	ME1P	0.990	**	1	0.907	0.933	n.s.
Mean Head Width	FL1P	0.978	**	1	0.900	0.920	n.s.
	ME1P	0.987	**	1	0.907	0.932	n.s.
Mean Head Depth	FL1P	0.981	**	1	1.013	1.032	n.s.
	ME1P	0.982	**	1	1.03	1.049	n.s.
Mean Fore Limb Length	FL1P	0.981	**	1	1.049	1.058	n.s.
	ME1P	0.991	**	1	1.048	1.058	n.s.
Mean Hind Limb Length	FL1P	0.995	**	1	0.924	0.928	n.s.
	ME1P	0.992	**	1	0.93	0.938	n.s.
Adult life span	FL1P	0.993	**	---	1.044	1.051	---
	ME1P	0.993	**	---	1.041	1.048	---
SVL at maturity	FL1P	0.999	**	1	0.97	0.980	n.s.
	ME1P	0.999	**	1	0.95	0.953	n.s.
Clutch size	FL1P	0.989	**	0	1.665	1.683	**
	ME1P	0.999	**	0	1.652	1.668	**
(+)	FL1P	0.973	**	0	0.480	0.493	*
	ME1P	0.977	**	0	0.486	0.498	*
Hatchling SVL	FL1P	0.946	*	1	0.08	0.845	n.s.
	ME1P	0.94	**	1	0.841	0.894	n.s.
Hatchling mass	FL1P	0.945	*	3	1.833	1.939	*
(+)	ME1P	0.938	**	1	0.547	0.583	**

*r* = correlation coefficient; P*_r_*: significance of correlation coefficient; b_exp_: expected value of the regression slope under isometry relationship; b_OLS_: slope of ordinary least squares (OLS) regression; b_RMA_: slope of the reduced major axis (RMA) regression; P_RMA_: *p* value of the difference between b_exp_ and b_RMA_. (+): Female BW as independent variable. *: *p* < 0.05; **: *p* < 0.01; ***: *p* < 0.001.

## Data Availability

Restrictions apply to the availability of these data. Data were obtained directly from previous works published by authors and collaborators. Data reported in the current review are not deposited in any repository. However, specific data may be available from the authors on request.
